# Analysis of the technological production of three professional master’s programs in the field of nursing*

**DOI:** 10.1590/1518-8345.3916.3276

**Published:** 2020-06-01

**Authors:** Rejane Eleuterio Ferreira, Cláudia Mara de Melo Tavares

**Affiliations:** 1 Universidade Federal Fluminense, Escola de Enfermagem Aurora Afonso Costa, Niterói, RJ, Brazil.

**Keywords:** Education, Nursing, Graduate, Education, Professional, Scientific Research and Technological Development, Nursing, Applied Research, Health Postgraduate Programs, Educação de Pós-Graduação em Enfermagem, Educação Profissionalizante, Pesquisa Científica e Desenvolvimento Tecnológico, Enfermagem, Pesquisa Aplicada, Programas de Pós-Graduação em Saúde, Educación de Posgrado em Enfermería, Educación Profesional, Investigación Científica y Desarrollo Tecnológico, Enfermería, Investigación Aplicada, Programas de Posgrado en Salud

## Abstract

**Objective:**

to analyze the technological production of three professional master programs in the area of Nursing.

**Method:**

documentary research on primary sources. A total of 100 graduate nurses from three professional master’s programs in Nursing in the Southeastern region of Brazil were analyzed, based on the following variables: time of training, typology of products; context of products and technological and educational classification, welfare and management. The study was guided by the question: “How is the technological production of the dissertation of the professional master’s degree according to typology, context and technological classification? The analysis of the data was based on the construction of a table that categorized the products according to the typology.

**Results:**

development of techniques was the main type of product found, being expressed by flowcharts, protocols, guidelines and training courses. The products were mostly developed in the hospital context, in the educational technology format.

**Conclusion:**

the technological production of the analyzed programs is restricted, diffuse and, in its majority, of low social impact and has no relevance in the body of the dissertation. Despite the wide possibility of conclusion work, the dissertation, accompanied by an article and/or technical productions, is the main form of presentation.

## Abstract (pt)

**Objetivo:**

analisar a produção tecnológica de três programas de mestrado profissional na área da Enfermagem.

**Método:**

pesquisa documental em fontes primárias. Foram analisados 100 trabalhos de conclusão de curso de enfermeiros egressos de três programas de mestrado profissional na área da Enfermagem, da região Sudeste do Brasil, a partir das seguintes variáveis: tempo de formação, tipologia dos produtos; contexto dos produtos e classificação tecnológica-educacional, assistencial e gerencial. Norteou-se o estudo pela questão: “Como se caracteriza a produção tecnológica dos trabalhos de conclusão do curso de mestrado profissional segundo a tipologia, o contexto e a classificação tecnológica?”. A análise dos dados deu-se a partir da construção de um quadro que categorizou os produtos de acordo com a tipologia.

**Resultados:**

desenvolvimento de técnicas foi o principal tipo de produto encontrado, sendo expressos por fluxogramas, protocolos, diretrizes e cursos de capacitação. Os produtos foram desenvolvidos, em sua maioria, no contexto hospitalar, no formato tecnologia educacional.

**Conclusão:**

a produção tecnológica dos programas analisados é restrita, difusa e, em sua maioria, de baixo impacto social e não possui relevo no corpo da dissertação. Apesar da ampla possibilidade de modalidades de trabalho de conclusão, a dissertação, acompanhada de artigo e/ou produções técnicas, é a principal forma de apresentação.

## Abstract (es)

**Objetivo:**

analizar la producción tecnológica de tres programas de maestría profesional en el área de enfermería.

**Método:**

investigación documental en fuentes primarias. Fueron analizados 100 trabajos de conclusión de curso de enfermeros graduados de tres programas de maestría profesional en el área de enfermería, de la región sureste de Brasil, a partir de las siguientes variables: tiempo de formación, tipología de los productos, contexto de los productos y clasificación tecnológica educacional, asistencial y administrativa. Este se orientó por la pregunta: “¿Cómo se caracteriza la producción tecnológica de los trabajos de conclusión del curso de maestría profesional, según la tipología, contexto y clasificación tecnológica?”. El análisis de los datos se realizó a partir de la construcción de un cuadro que categorizó los productos de acuerdo con la tipología.

**Resultados:**

el desarrollo de técnicas fue el principal tipo de producto encontrado, siendo expresado por diagramas de flujo, protocolos, directrices y cursos de capacitación. Los productos fueron desarrollados en su mayoría en el contexto hospitalario, en el formato tecnología educacional.

**Conclusión:**

la producción tecnológica de los programas analizados es restringida, difusa y en su mayoría de bajo impacto social y no posee relevancia en el cuerpo de la disertación. A pesar de la amplia posibilidad de modalidades de trabajo de conclusión, la disertación, acompañada de artículo y/o producciones técnicas, es la principal forma de presentación.

## Introduction

The professional master’s degree (PM) is a s*tricto sensu* post-graduation modality aimed at training professionals in various fields of knowledge through the study of techniques, processes or themes that meet the de\mands of the labor market^(^ 1 ^)^.

As an expected product, the PM, in addition to the master’s degree training, implies the presentation of a scientific investigation involving topics in the student’s area^(^ 2 ^)^. However, one cannot only study the problems arising from professional practice; one must, from these studies, present possible solutions translated into various types of technological production^(^ 3 ^)^.

Technological/technical production is considered to be that produced by a permanent teacher and student, which is not characterized as scientific production, being recognized by the interaction processes between academia and society^(^ 4 ^)^.

This product is what most differentiates it from other s*tricto sensu* graduate programs, since the student develops, in addition to a degree work, technological productions that can be different forms of products and services, whose application results in improvements in the health of the population^(^ 2 ^)^. In this way, it must be considered that not only the professional is the object of formation, but the service itself is also the subject and motive of the formative process^(^ 5 ^)^.

For Nursing, the PM is characterized as a potentiality to improve care, management, education and research itself because the courses are based on principles such as applicability, flexibility, organicity, innovation and appreciation of professional experience^(^ 4 ^)^.

However, the PM is still establishing its specificities, especially in the expertise in technological production and innovation, seeking a better understanding of the elaboration of its products, as conclusion works, so that the applicability of these, in fact, contributes to the quality of health services^(^ 5 ^)^. The technical production was evaluated for the first time in the last four-year evaluation of PM programs (2013-2016) and is a challenge for both the programs and the Coordination for the Improvement of Higher Level Personnel (CAPES)^(^ 4 ^)^.

In this sense, the objective of this study was to analyze the technological production of three professional master programs in the area of Nursing.

## Method

This is a documentary search on primary sources, i.e. documents that have not received scientific treatment^(^ 6 ^)^. In this study, the conclusion of three professional master’s programs in Nursing, in the Southeastern region of Brazil were analyzed. For a better understanding of the data, the programs will be referred, throughout the article, with the letters A, B and C.

As criteria for inclusion of the study sample, work was defined to conclude the course of nurse and the professional master’s degree that obtained the title during the evaluation period of the last quadrennium of CAPES (2013 to 2016). The exclusion criteria established were: course completion works that have products under secrecy and/or in patent process and other non-nursery professionals, since two courses are multi-professional.

The search was carried out from November 2017 to January 2018 and was guided by the following question: How is the technological production of the conclusion works of the professional master’s degree according to typology, context and technological classification?

The survey started with a survey at the program office after the coordinators’ agreement. The following information was accessed: full name of the egresses; date of the beginning of the course; date of the final defense; title of the work and form of access to the works of conclusion of the course. This information was organized in an Excel spreadsheet for a better visualization of the data.

It was found, considering the time cut of the study, that during the last four-year evaluation of CAPES (2013 to 2016), there were 154 defenses. Following the exclusion criteria, 27 conclusion works by non-nursery professionals were disregarded. 127 papers were selected for analysis, but 27 were not available for consultation, because the egresses did not present the final version in the course.

Access to the work was given in two ways: two programs make available the research carried out in the institutional repository online and another program, in printed form, in the files available at the course office.

The data was collected through a structured script, which allowed obtaining information on the following variables: time of formation of students; typology of products; context of products and technological classification - educational, welfare and management.

As a parameter to classify the typology of the products, an instrument to classify the technical production of the Nursing area of CAPES was used, four years from 2013-2016^(^ 4 ^)^.

The CAPES instrument evaluates technological productions along the following axes: patent-generating or off-patent product; training and continuing education; dissemination of production and specialized technical services^(^ 4 ^)^. This study will use only the product axis that may or may not generate patents.

The products are characterized by the development of technical or technological product, susceptible or not of protection, being able to generate registrations of property of patents, intellectual production and/or copyrights. The following are considered possible types of technical production: application development; patentable product/process development; technical development; educational and instructional material development; social technology development; unpatentable process/technology development^(^ 4 ^)^.

The context of product development was evaluated based on the scenario where the product could be deployed and classified by areas of care, according to the Portfolio of Services - City Hall of Rio de Janeiro (2016)^(^ 7 ^)^, training area and other fields.

The areas of care are: Primary Care - Municipal Health Center (MHC), Family Clinic (CM); Secondary Care - Polyclinic, Center for Psychosocial Care (CAPS), Emergency Care Unit (ECU) and Rehabilitation Center; Tertiary Care - Maternity, Hospital, Institute^(^ 7 ^)^.

The following areas were considered for training: technical vocational course; undergraduate; post-graduate l*ato sensu* and *stricto sensu*.

To identify which type of technology was being developed in the Master’s degree, the technological classification was used as a reference: educational, welfare and managerial^(^ 8 ^)^.

Educational technologies are the systematic set of scientific knowledge that allows planning, executing, controlling and accompanying the formal or informal educational process and, thus, favoring the construction and reconstruction of knowledge (primers, brochures, videos). Assistance technologies, on the other hand, are the set of technical-scientific knowledge systematized, procedural and instrumental, which enables the promotion of the quality of health care to the customer (theories and scales). And the management technologies are the set of theoretical and practical actions to manage health actions and services, whose objective is to intervene in professional practices in order to improve their quality (manuals, institutional routines, welcome and bonding)^(^ 8 ^)^.

These contents were organized in Excel spreadsheet. The analysis of the data was based on the construction of a table that categorized the products according to typology, context and technological classification: educational, welfare and managerial.

It is worth noting that the documentary research used information from the public domain and, because it did not involve human beings directly in the collection of the data, there was no requirement for approval from the Research Ethics Committee^(^ 9 ^)^. The evaluation of the works and the characterization of the products kept the anonymity of the authors and institutions.

After the selection and preliminary analysis of the documents, the data was analyzed. In this stage of document analysis, knowledge and ways of understanding the phenomenon under study were reworked, determining trends, taking into account the theme or the initial questioning and, as far as possible, making the inference^(^ 6 ^)^. The results were discussed, following the instructions of the method, in the light of the CAPES legislation on professional master’s degrees and related national scientific literature.

## Results

The results were discussed, following the instructions of the method, in the light of the CAPES legislation on professional master’s degrees and related national scientific literature.

Table 1 shows the typology of the products of Master’s dissertations in programs A, B and C.

**Table 1:** Typology of products, of Master's dissertations in programs A, B and C. Rio de Janeiro, RJ, Brazil, 2018

Type	Subtypology	Programs			Total (n)*
		A	B	C	
Application Development	Chat, fanpage, software, prototype, video, application, realistic simulator	6	9	4	19
Patentable product/process development		0	1	0	1
Technical development	Flowchart, form, script, protocol, checklist, Terminology Subgroup ICNP^†^, Nursing diagnosis proposal to NANDA^‡^, guidelines, protocol validation, training course and workshop	24	28	9	61
Development of teaching and instructional material	Games, manuals, brochures, multimedia and educational portal	1	11	1	13
Development of social technology	Forum and Regional Meeting	2	0	0	2
Development of non-patentable	Management processes: evaluation tool	0	3	1	4

* N = Absolute number;

† ICNP = International Classification for Nursing Practice;

‡ NANDA = North American Nursing Diagnosis Association.

Technical development was the most produced type of product in the group, with 61%, followed by application development, with 19%, and didactic material development, with 13%.

Table 2 shows the context where the products of the Master’s dissertations in programs A, B and C were developed.

**Table 2:** Context of the products of Master's dissertations in programs A, B and C. Rio de Janeiro, RJ, Brazil, 2018

Classification	Program A	Program B	Program C	Total (n)
Primary care	11	2	0	13
Secondary care	2	6	1	9
Terciary care	6	42	13	61
Qualification	14	0	0	14
Others	0	2	1	3

It was found that tertiary care was the main context for the construction of technological productions, predominantly in the hospital area. The training area was the second largest production context related mainly to the *lato sensu* training in residency courses. In the sequence, it has the primary care as the main scenario the Family Health Strategy (FHS) and, finally, the secondary care, having as scenario the ECU. The other contexts were supervisory bodies and the Federal Nursing Council(COFEN).

Table 3 shows the products of the Master’s dissertations in programs A, B and C, according to the technological classification: educational, welfare and managerial.

**Table 3:** Classification of products developed by professional master's degree nurses according to technological classification: educational, welfare and managerial. Rio de Janeiro - RJ, Brazil, 2018.

Classification	Program A	Program B	Program C	Total (n)
Educational	29	26	2	57
Assistential	0	17	8	25
Managerial	4	9	5	18

It was found that the main technology developed was educational. Among them are: training course; workshop; reflection group; video; virtual environment and booklets. Tertiary care was also the main context for the development of assistance and management technologies, and the main technological productions were: protocol, guidelines and flow chart. Programs B and C have developed three managerial technologies for COFEN: assessment tool, monitoring roadmap and professional sizing calculation.

## Discussion

Initially, it is important to emphasize the difficulty encountered to identify and classify the typology of the products, being necessary to return to the material in its entirety, sometimes, for a better understanding and, consequently, to classify it correctly, because, many times, the author did not know how to determine the typology of the product or the classification was not consistent with the material presented.

This aspect deserves attention, since scientific articles and national forums still discuss the identity of the PM, the technological production developed in the programs, the implementation process and its consolidation, these being the main aspects to be resolved^(^ 10 ^)^.

In the last four-year evaluation report, the evaluation committee stressed the need for improvements in the quality of technical production records, since the dispersion hinders the overall analysis of the performance of the programs and of the area itself^(^ 4 ^)^. The mishandling in the recording of technical productions may reflect the lack of readability of some end-of-course papers.

To improve data and information on the Sucupira Platform, a platform where technical outputs and all evaluation items of the Master’s program are recorded, the Area Coordination and the Advisory Committee prepared, in 2012, an orientation script for records in the descriptive fields of CAPES Collection, which has been updated and made available to program coordinators^(^ 4 ^)^.

It must be considered that, until the year 2012, there was no technological Qualis for the evaluation of PM programs. Such evaluation was carried out following criteria and metrics of the academic master’s degree (AM), with strong emphasis on intellectual production^(^ 11 ^)^. Qualis technology was used for the first time in the four-year assessment 2013 to 2016^(^ 4 ^)^, which may justify the difficulty of recording the technical productions pointed out in the evaluation report.

In the last four years, the books produced by the area (Qualis books) were incorporated into the evaluation process, fruit of discussions and contributions with peers in seminars and forums in the area^(^ 12 ^)^.

Although there are advances in the process of evaluating professional master’s degrees, the publication of articles in well-classified journals in Qualis has even greater value within the *stricto sensu* post-graduate program. It is believed that this panorama will not change until the technical production is valued on an equal footing with the academic production, so-called intellectual^(^ 11 ^)^.

The technical productions developed in the MP were included in a specific chapter of the dissertations analyzed. Sometimes, the product constituted the technique developed throughout the research - as a group, a workshop, a course. Other times, the product was presented as a physical material - folder and booklet, although these were not attached to the dissertation.

In general, the technical productions had the following information: modality; product description; objectives; target audience and, sometimes, product image. The term “in general” was used because the products were not organized in standardized format. The similar point observed in all programs was an attached article at the end of the dissertations.

It should be noted that Ordinance No. 17, 2009, established different formats for the Final Conclusion Work of the Professional Master’s Course, which go beyond the dissertations, an exclusive model of academic modalities, such as: systematic and in-depth review of literature; article; patent; intellectual property records; technical projects; technological publications; development of applications, teaching and instructional materials and products, processes and techniques; production of media programs; publishing; final research reports; software and case studies^(^ 13 ^)^.

Despite this range of possibilities, the PM’s conclusion papers are still presented in the classic academic dissertation model. This structure, accompanied by an article and/or technical product, has already been evidenced in a study that highlighted the difficulties in overcoming the academic model^(^ 14 ^)^. Another study^(^ 15 ^)^ verified the academic influence in the PM’s works through the similarity in the adoption of the verbs that conduct the objectives of the investigations, as well as the methodological designs adopted, highlighting the need for the PM to have its own identity in the construction of the product. It is also important to emphasize the difficulty found in the PM for the student’s research to be accommodated in the dissertation model^(^ 16 ^)^.

A recently published study, which evaluated the proposals and dissertations of the AM (academic master’s degree) and PM courses, presented results similar to those of the studies presented in the previous paragraph, concluding that the significance of the PM and the training of professionals who make use of science to better qualify their work do not seem to distinguish it from the AM, on the contrary, they are exactly the point in common between one and the other modalities^(^ 5 ^)^.

For these results, three hypotheses are formulated: 1) teachers would not dominate another form of final work presentation; 2) as the AM of the institution advocates the elaboration of products of this nature, the proposals of PM only repeated what was established; 3) the idea still largely crystallized that the PM, to have the same value as the AM, must require the same product^(^ 16 ^)^.

The three hypotheses show that there is still a strong influence of academic programs on the outcome of professionals associated with the lack of clarity on the principles of PM permeating the Brazilian graduate school. This is to the detriment of the perception of Higher Education Institutions (HEIs) about the PM’s potentiality in its social mission. Hence the low valuation of this type of post-graduate courses^(^ 14 ^)^. CAPES itself, in one of its reports in 2016, admits that the PM is still impregnated with academic criteria^(^ 4 ^)^.

Today, Ordinance nº60 of 2019 is in force, which regulates the professional master’s and doctorate in the scope of CAPES and which will guide the evaluation of the programs of the quadriennium in activity. This ordinance states that the conclusion work must meet the demands of society, aligned with the objective of the program, using the scientific method and the state of the art of knowledge, following the principles of ethics, and that the regulation of the professional program must indicate the formats of the conclusion work, as well as the mechanisms of documented registration on the knowledge generated by the research, for verification and evaluation purposes. It is important to note that the document does not mention the types of work, although a technological Qualis is in the final stage of preparation by CAPES^(^ 17 ^)^.

The program performance assessment form in the 2013-2016 quadrennium analyzed five items: proposal; teaching staff; student body, theses and dissertations / conclusion papers; intellectual production and social insertion. Os quesitos de avaliação têm indicadores qualitativos e/ou quantitativos com estratificações de cortes para esses conceitos, sendo eles: Insuficiente; Fraco; Regular; Bom ou Muito Bom, de forma a permitir sua classificação segundo as notas 1, 2, 3, 4 ou 5^(^ 4 ^)^.

The technical production of the PM was evaluated in terms of intellectual production, using indicators, quantifying the technical production of the program (item 4.2 of the evaluation form) and its distribution among permanent teachers (item 4.3 of the evaluation form). This assessment provides a global view of the significance of this type of production in the area^(^ 4 ^)^.

Among the types of technical productions of the PM, it was found, in the study on screen, a greater production in the typology of development of techniques, including protocols, flowcharts, forms and training activities. These types of products are worth a grade of five to ten points, according to the CAPES evaluation framework. It can be deduced, taking into account that the score of technical productions ranges from one to 50 and that only one product in this study could receive the maximum score, as it is a patentable product, that the evaluation of technological productions developed in courses of PM studied does not propose to reach the maximum score.

The aforementioned fact is confirmed through the CAPES evaluation report, as two of the programs studied received a grade 3, meeting the minimum quality standard, and one received a grade 4, considered to have performed well in the last quadrennium. Regarding the impact of technological production, two programs were evaluated with regular impact and one, with good impact and are still presented in small numbers and with greater impact at the local and regional level^(^ 4 ^)^.

For the next quadrennial evaluation 2021, the number of items on the evaluation form was reduced from five to three: program, training and impact on society^(^ 18 ^)^.

With regard to the Program, the aim is to evaluate the functioning, structure and planning of the graduate program in relation to its profile and objectives. The programs’ self-assessment will be considered in the next four-year assessment with the objective of seeking to value training and its process, not only focusing on the product. The item Training will focus on the quality of trained human resources. The analysis will cover aspects such as the quality of theses, dissertations, the intellectual production of students and teachers and research activities, as well as the evaluation of the graduate. Whereas the Impact on Society will be related to the impacts generated by the training of human resources and, the production of knowledge for the program. The evaluation will verify the innovative character of intellectual production, the economic and social effects of the program, internationalization and visibility^(^ 18 ^)^.

This form was applied at the mid-term seminar that took place in August 2019. The final version will be used in the evaluation of the current quadrennium. It is believed that the new form is quite solid, robust, to provide a more simplified and clear evaluation for the academy as a whole, although there are not yet, in the form, the questions regarding technological production. The expectation is that this new sheet may be a transition to a multidimensional evaluation^(^ 18 ^)^.

As for the context and type of technology developed in the PM, it was found that the main technology developed was educational. The program focused on education developed 14% in the context of vocational training and 15% in the areas of care, with a focus on continuing and continuing education.

However, two programs focused on nursing care and hospital technology developed 28% of the educational technologies analyzed here, all of them in the areas of care, with greater production in tertiary care, and with objectives correlated to continued and permanent education.

Increasingly, nursing professionals are making use of educational technologies to provide care^(^ 19 ^)^. Educational technologies intermediate health education actions and facilitate the relationship between professionals and the patient, favoring access to information together with emotional support and risk assessment, and enabling reflections on values, attitudes, behaviors and strategies to live with the disease^(^ 20 ^)^.

In this sense, continuing education has a strong contribution, since it occurs from the identification of daily problems and needs of services and users of the health system, which is involved with the responsibility to improve the health conditions of the population^(^ 21 ^)^.

Tertiary care was also the main context for the development of assistive and managerial technologies. One justification for the great concentration of research in this context is that one of the courses focuses on technology in the hospital space.

The hospital area was the main scenario for the study and development of technological production of PM. This shows that, in addition to assistance, hospitals are also spaces for education, training of human resources, research and evaluation of health technologies for the Health Care Network, provided for in the National Policy for Hospital Care (NPHC) under the Single Health System (UHS), in Ordinance No. 390, 2013^(^ 22 ^)^.

The training area was the second most productive context related, mainly, to the *Lato sensu* training in the residence courses, however, this result happened because one course has its focus on health teaching and the others did not present research in this context in the time cut studied.

In such a context, the integration of teaching and service is an important proposal to consolidate the processes of change in the training of health professionals. This integration enables the reduction of the theory-practice dichotomy, brings students/professionals closer to UHS principles and assists services in the development of actions and training of professionals, improving the quality of care^(^ 23 ^)^.

It is added that for the more solid training of health professionals, education and work are articulated through the insertion of both in the care network. These are unique moments in which actions are imbricated and of mutual influence^(^ 23 ^)^.

The third context with the greatest product development was primary care, with FHS as the main scenario - researched mainly by students of the course focusing on health education. Primary health care is the gateway to the Brazilian health system and has a strong impact on the other areas of care^(^ 24 ^)^. This context has become the main learning scenario, with emphasis on active learning methods. Active methodologies have been the focus of interest in Nursing research as the use of these methods favors meaningful learning, the construction of knowledge, in addition to developing, in the professionals, skills and attitudes, with autonomy and responsibility^(^ 23 ^)^.

Secondary care is also contemplated, but with fewer reports, taking the ECU as a scenario. Another context considered was the Federal Council of Nursing (COFEN), for which products related to the assessment instrument were developed.

The literature suggests that, although there are challenges to be faced, health master’s programs contribute positively to the academic and professional milieu^(^ 25 ^)^. Nursing has been gaining prominence in the application of technologies, with opportunities to implement and/or develop them for further growth of the profession and benefits of the relationship between professional and customer^(^ 19 ^)^.

This study presented a synthesis of the technological productions developed in the scope of the professional Master in Nursing in the Southeast region of Brazil. The results pointed out some observations of the technological productions that deserve a better evaluation of the programs in order to strengthen even more the professional master’s degree that, due to its assumptions, can give more agile answers to the implementation of public health policies in the country.

The temporal and regional cut-off can be considered as a limitation of this study. The documentary research does not allow the evaluation of the implementation and impact of technological productions in the practical assistance, managerial and educational field, indicating the importance of the theme and the need for new studies.

However, the results of this research give visibility to the technological productions coming from the PM programs in the area of Nursing and reveal pertinent information, being able to contribute with the evaluation bodies that are in the moment of reconfiguration of the evaluation instruments. The other programs may benefit from this study, taking some parameters to review their technological productions, improving their design, form of presentation and social relevance.

## Conclusion

The analysis of the products of the professional Master of Nursing in the Southeast region of Brazil shows the development of techniques - flowcharts, protocol, forms and training course, as the main typology resulting from the course conclusion work. Most of these products were developed in the hospital context and in the educational technology format, thus referring to improvements in the working process of the nurse.

It is worth mentioning the difficulty to identify the technological productions from the course conclusion works presented in the analyzed cutout. The technological production proved to be restricted, diffuse and in its majority of low social impact, besides not appearing prominently in the body of the dissertation.

Despite the countless possibilities of working methods for the completion of professional master’s degrees, the dissertation, accompanied by an article and/or technical productions, is the main form of final work presentation.
